# High-degree atrioventricular block. An unusual presentation of Takotsubo cardiomyopathy: a case report

**DOI:** 10.1186/s43044-021-00144-x

**Published:** 2021-02-25

**Authors:** Pablo Revilla-Martí, Juan F. Cueva-Recalde, Jose A. Linares-Vicente, Sara Río-Sánchez, Jose R. Ruiz-Arroyo

**Affiliations:** Cardiology Department, Hospital Clínico Universitario “Lozano Blesa”, Av. San Juan Bosco 15, 50009 Zaragoza, Spain

**Keywords:** Atrioventricular block, Acute coronary syndrome, Takotsubo cardiomyopathy

## Abstract

**Background:**

Takotsubo cardiomyopathy is a non-ischemic cardiomyopathy characterized by acute left ventricular systolic dysfunction with transient wall motion abnormalities without a culprit coronary stenosis or other concurrent diagnoses. Its coexistence with transient high-degree AV block is very infrequent.

**Case presentation:**

A 61-year-old man presented with a new onset of high degree AV block without ST segment deviations developing an anterior and apical dyskinesia with a low left ventricular ejection fraction in the absence of coronary artery disease.

**Conclusion:**

Atrioventricular block is an uncommon presentation of Takotsubo syndrome. The management of patients with relevant conduction disorders in this scenario is a challenge for the clinician. In case of persistence of advanced conduction disorders, it seems appropriate to implant a pacemaker.

## Background

Takotsubo cardiomyopathy (TCM) is characterized by transient left ventricular dysfunction in the absence of coronary artery disease. It has a clinical presentation usually resembling an acute coronary syndrome, and patients will present with sudden acute chest pain or dyspnea and ST segment elevation or T wave inversion on the electrocardiogram. However, its presentation as high-degree atrioventricular (AV) block is rare [1, 2].

## Case presentation

**Fig. 1:**
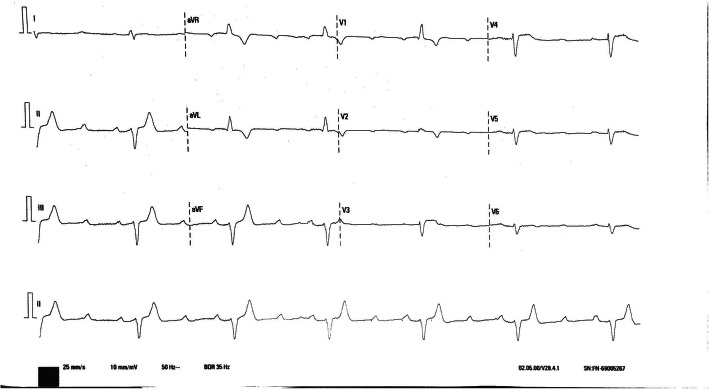
A 12-lead electrocardiogram showing sinus rhythm with high-degree atrioventricular block

A 61-year-old man with a history of chronic obstructive pulmonary disease was hospitalized for rapid onset dyspnea. On initial evaluation at the emergency department, the patient was afebrile with an initial blood pressure of 90/50 mmHg and blood oxygen saturation level of 85%. He presented with bradycardia, and the electrocardiogram showed new onset of high-degree AV block without ST segment deviations (Fig. 1). A marginal high-sensitive troponin elevation and a normal transthoracic echocardiogram were evidenced. Nevertheless, 3 h later, troponin levels increased significantly, and a new transthoracic echocardiogram showed apical dyskinesia with a left ventricular ejection fraction (LVEF) of 30%.

**Fig. 2:**
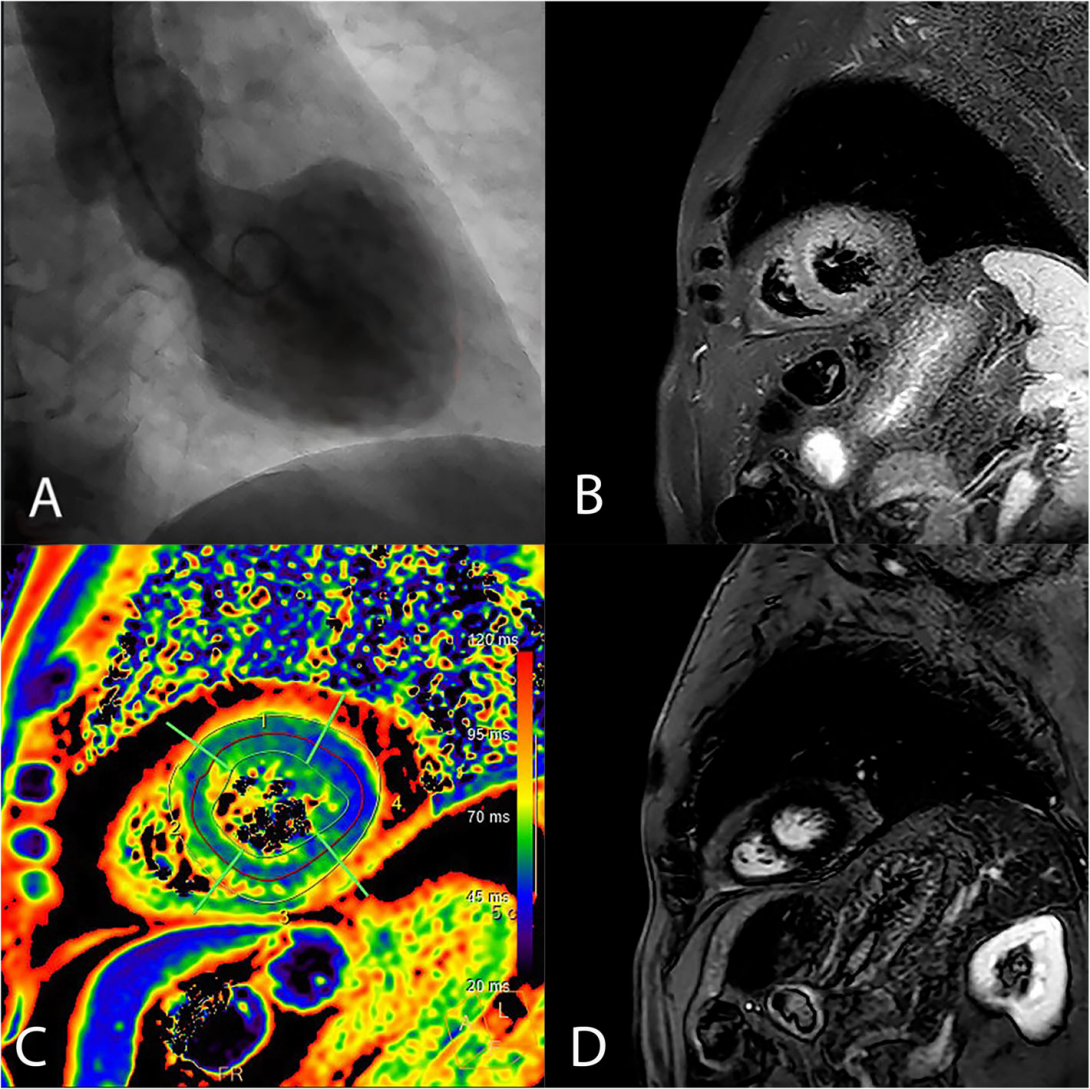
**a** Ventriculography showing mid-anterior, antero-apical, and inferoapical dyskinesia. **b** Cardiac magnetic resonance. T2 STIR sequence shows high signal intensity in the apical septum wall. **c** T2 mapping. Abnormal T2 apical septum and inferior apical (66 –62 ms). **d** Phase-sensitive inversion recovery sequence showing no late gadolinium enhancement

An emergent coronary angiogram was performed which reveal normal epicardial coronary arteries with a left ventriculography (Fig. 2a) showing mid-anterior, antero-apical and inferoapical dyskinesia, hypercontractility of the basal segments, and an estimated LVEF of 30%. During cardiac catheterization, the patient recovered normal AV conduction. After initial intravenous diuretics treatment, the following days, he remained asymptomatic with an echocardiography and a cardiac magnetic resonance performed 7 days after the admission showing a normal LVEF and apical edema in T2 stir sequences (Fig. 2b, c) without late gadolinium enhancement (Fig. 2d). According to the INTERTAK criteria, TCM diagnosis was made [1, 2].

## Discussion

TCM is a rare disease, and its coexistence with transient high-degree AV block is very infrequent. AV block is reported in 2.9% of the total cases of TCM, and its relationship with TCM has not been clarified yet [3]. The physiopathology is difficult to explain as TCM is supposed to have a magnified sympathetic tone and the apex, far away from the AV node, is the location of the most common wall segment motion abnormality. Diffuse spams in small coronary branches causing ischaemia and an increase in vagal tone are the hypothesized mechanisms of AV conduction disturbance [4].

The recovery of left ventricular dysfunction is not necessarily linked to the resolution of the AV conduction disturbances, suggesting different pathophysiologic mechanisms. In fact, in the majority of cases, AV block persists once the left ventricular function is normalized leading to a permanent pacemaker implantation. The indication and timing to place a permanent pacemaker is still a quandary, and decision should be individualized based on the lack of the conduction improvement or significative findings in the electrophysiological study [5, 6].

Although data from case series report that the majority of patients receive a permanent pacemaker [6], in our patient, due to the unexpected fast recovery of the normal AV conduction, even before of the left ventricular function normalization, we did not implant any device. Very few cases describe the normalization of AV conduction related to TCM [7–9], and this is the first case that reports it just a few hours after admission. Recent data suggest that advanced atrioventricular block is not associated with worse in hospital outcomes unlike what occurs with ventricular arrhythmias [10]. Two years after the admission, the patient remains asymptomatic with a first-degree AV block on the electrocardiogram.

## Conclusion

AV conduction disturbances are a rare presentation of Takotsubo cardiomyopathy, and the management of these patients is still challenging. In case of persistence of AV block after recovery of LVEF, permanent pacemaker implantation should be considered.

## Data Availability

Data sharing is not applicable to this article as no datasets were generated or analyzed during the current study.

## Abbreviations

AV: Atrioventricular
LVEF: Left ventricular ejection fraction
TCM: Takotsubo cardiomyopathy
